# Dopaminergic Drugs Modulate Resting-State EEG Microstates in Healthy Participants

**DOI:** 10.1007/s10548-026-01232-4

**Published:** 2026-07-15

**Authors:** Renate de Bock, Amatya J. Mackintosh, Alexandra Korda, Alina Preuss, Daniel J. Hauke, Andreea O. Diaconescu, Philipp Sterzer, Stefan Borgwardt, Christina Andreou

**Affiliations:** 1 Department of Psychiatry (UPK), University of Basel, Wilhelm Klein-Strasse 27, Basel, 4002 Switzerland; 2 Faculty of Psychology, Department Clinical Psychology and Epidemiology, Missionsstrasse 62a, Basel, 4055 Switzerland; 3 Translational Psychiatry, Department of Psychiatry and Psychotherapy, University of Lübeck, Ratzeburger Allee 160, 23562 Lübeck, Germany; 4 Center of Brain, Behavior, and Metabolism (CBBM), University of Lübeck, Ratzeburger Allee 160, 23562 Lübeck, Germany; 5 Hawkes Institute, Department of Computer Science, University College London, 90 High Holborn, London, WC1V6LJ UK; 6 Krembil Centre for Neuroinformatics, Centre for Addiction and Mental Health, 250 College St, Toronto, ON M5T1R8 Canada; 7 Department of Psychiatry, University of Toronto, Toronto, Canada

**Keywords:** Electroencephalogram, Resting-state, EEG microstates, Dopamine, L-DOPA, Haloperidol

## Abstract

Changes in the temporal parameters of EEG microstates are observed in various psychiatric illnesses, including psychotic disorders. These changes have also been observed in individuals with a clinical or genetic high risk for psychosis. It is unclear whether dopamine abnormalities, distinctive of psychotic disorders, contribute to microstate alterations. Fifty-eight healthy participants (M: F = 32:26) took part in a single-dose drug challenge study. Using a randomized, double-blind, cross-over, and placebo-controlled design, participants received the dopamine precursor L-DOPA (100 mg), the dopamine antagonist haloperidol (2 mg), and a placebo. Resting-state (10 min, eyes-closed) 64-channel EEG was recorded at peak drug effects. We calculated temporal parameters (coverage, duration, and occurrence) of 5 microstate classes (A-E) and compared them between drug conditions using linear mixed-effect models. Significant group x class interactions were observed for all parameters. Compared to placebo, parameters of microstate B were decreased under L-DOPA (duration and occurrence) and haloperidol (coverage, duration, and occurrence). Coverage and occurrence of microstate C were reduced under L-DOPA compared to haloperidol and placebo. Parameters of microstate E were reduced under L-DOPA compared to haloperidol (coverage and occurrence) and placebo (coverage, duration, and occurrence). Both haloperidol and L-DOPA increased all parameters of microstate D, compared to placebo. Our results show that resting-state EEG microstates are modulated by dopaminergic drugs. However, extrapolations from healthy controls to patients with psychotic disorders are complicated by various factors. Further studies should investigate the (long-term) effects of haloperidol and other antipsychotics on EEG microstates in patients with psychotic disorders.

**Supplementary Information:**

The online version contains supplementary material available at https://doi.org/10.1007/s10548-026-01232-4.

## Introduction

Resting-state EEG microstates analysis is a robust method to study large-scale network dynamics in humans. Almost half a century ago, Lehmann et al. (1987) discovered that certain configurations, or topographies, of scalp potentials are temporally stable (30–120 milliseconds) before rapidly shifting to the next configuration. Since then, several studies have consistently identified the same 4–7 topographies of microstate classes (Tarailis et al. 2023). These topographies are present across different age groups (Bagdasarov et al. 2024; Koenig et al. 2002) and show both short-term (average ICCs = 0.874–0.920) and long-term test-retest reliability (average ICCs = 0.671–0.852) (Kleinert et al. 2023). After identifying the topographies in an EEG-dataset, the microstate analysis procedure enables the extraction of temporal parameters: the percentage of time coverage of each microstate, the average duration in milliseconds, and the occurrence rate per second. The parameters are then used to compare and/or characterize different demographical groups, and/or clinical conditions.

Many resting-state EEG microstate studies in clinical conditions were carried out in patients with psychotic disorders and schizophrenia (Andreou et al. 2014a, b; da Cruz et al. 2020; de Bock et al. 2020; Iftimovici et al. 2023; Koenig et al. 1999; Liebrand et al. 2024; Sun et al. 2022). Meta-analyses have associated an increase of coverage and occurrence in microstate C, and a decrease of coverage and duration of microstate D with schizophrenia (da Cruz et al. 2020; Rieger et al. 2016; Vass et al. 2025). At the same time, individual studies also reported changes in all the other microstates classes, but those results are mixed. Some studies have linked changes in microstate parameters to psychotic symptoms such as hallucinations (Kindler et al., 2011), or negative symptoms (Giordano et al., 2018). However, the exact nature and pathogenesis of these alterations in patients with psychotic disorders remain unclear.

A prominent neurobiological model of psychotic disorders is the dopamine hypothesis (Howes and Kapur 2009), which postulates that dysregulation of dopamine neurotransmission plays a significant role in the emergence of psychotic symptoms. Indeed, antipsychotic drugs primarily function as dopaminergic D2 receptor antagonists, blocking or reducing the action of dopamine in certain brain regions (Kapur and Mamo 2003). A recent systematic review by our group indicated that aberrant dopamine activity might modulate resting-state functional connectivity (Mackintosh et al. 2021). However, the review did not include EEG microstates, and the precise impact of dopamine alterations on microstate dynamics remains unclear.

Pharmacological manipulation studies can provide more direct evidence on the relationship between changes in neurotransmitter activity and brain network dynamics. So far only one study investigated the effects of various antipsychotics (dopamine antagonists) on EEG microstates in healthy male participants (Yoshimura et al. 2007). The authors reported a heterogeneous pattern of results across different antipsychotics, with a significant increase of average total duration across classes after administration of haloperidol and chlorpromazine, and an increase of microstate D duration after administration of perospirone. However, the sample size was small, consisting of 14 only male participants.

Effects of opposing dopaminergic drugs have been reported to display both linear and non-linear (U-shaped) effects on fMRI resting-state connectivity (Cole et al. 2013). Linear effects along the dopamine continuum are suggested to be related to changes in dopaminergic activity underlying both the emergence of psychotic symptoms and their treatment (Howes and Kapur 2009). In contrast, non-linear effects of dopamine have been associated with cognitive processes (Cools and D’Esposito 2011). Cognitive symptoms represent a core feature of psychotic disorders and contribute substantially to functional outcomes (Lee et al. 2024; McCutcheon et al. 2023). Thus, to gain a complete understanding of dopaminergic effects, it is necessary to study dopaminergic drugs that both increase and decrease dopaminergic transmission.

The aim of this study was to investigate the acute effects of two dopaminergic modulators on resting-state EEG microstates in healthy participants. In a randomized, double-blind, cross-over design, participants received L-DOPA (a dopamine precursor), haloperidol (a dopamine D2 receptor antagonist), and placebo on three different days. We measured resting-state EEG and performed microstate analysis. Based on previous findings, we expected to find differences in most microstate classes. Drug effects that follow a linear pattern (from L-DOPA to placebo to haloperidol) could be relevant for psychotic disorders because they follow the same pattern of dopaminergic activity changes associated with symptom emergence and treatment. Drug effects that follow a non-linear (U-shaped) pattern are more likely to reflect unspecific symptom domains, such as cognitive impairment.

## Materials and Methods

The study was a double-blind, placebo-controlled, 3-way crossover trial assessing the effect of dopaminergic modulations on resting-state EEG microstates. The study was approved by the local ethics committee (Ethikkommission Nordwest- und Zentralschweiz, registration number 2016 − 01734), and all participants provided written informed consent.

### Participants

Eligible healthy individuals (aged 18–40 years) were recruited through the student website of the University of Basel, advertisements on the university’s online marketplace, and by word-of-mouth. Exclusion criteria were as follows: any past or current psychiatric or neurological disorders, a history of schizophrenia or bipolar disorder in a first-degree relative (Mini International Neuropsychiatric Interview; Sheehan et al. (1998), a history of cranio-cerebral trauma, arterial hypertension, cardiological or serious medical conditions posing a contraindication to the administration of L-DOPA or haloperidol, hypersensitivity reaction to any drug, pregnancy and breastfeeding, as well as current treatment with any (psychotropic or other) drug (including hormonal contraceptives). Participants received either a reimbursement (300 CHF) or a combination of monetary reimbursement and course credit (150 CHF + course credit). Participants were instructed in advance to adhere to a low-protein diet on the day of the session up to one hour before the session, and then refrain from any food, caffeine, and nicotine until the end of the session. Urine samples were collected at the start of each session to screen for drug use. Data collection took place at the research facilities of the University Psychiatric Clinics (UPK) and the Faculty of Psychology in Basel, Switzerland.

### Study Design and Procedure

Participants received capsules of either 100 mg L-DOPA and 25 mg benserazide (Madopar), 2 mg haloperidol (Haldol), or placebo. The dose of L-DOPA and haloperidol were chosen based on previous studies (Andreou et al. 2014a, b). The dose of haloperidol corresponds to a D2 receptor occupancy of around 70% (Kapur et al. 1997, 2000). Blood pressure and heart rate were monitored throughout the session to detect potential adverse reactions. The three sessions were separated by at least 7 days to allow a complete wash-out of the study drugs. The EEG recording was performed at the expected peak serum levels of each drug. This was achieved by using a double-dummy design (Table 1) to compensate for the different Tmax of L-DOPA and haloperidol. The study sessions for each participant took place around the same time of day (in the morning or afternoon).

**Table 1:** Double-dummy design of the study drug administration

Condition	t0	t1 (1.5 after t0)	t2 (2.5 h after t0)
L-DOPA	Placebo	Madopar	Start of the EEG session
Haloperidol	Haloperidol	Placebo	Start of the EEG session
Placebo	Placebo	Placebo	Start of the EEG session

### Sample Size Calculation

We calculated the sample size a priori using G*Power (Faul et al. 2007). We based the calculation on a single regression coefficient in a fixed linear model, with a Type I error probability of 5%, a power of 80%, and an effect size f2 = 0.15 (similar to the obtained effect size elsewhere (Andreou et al. 2014a, b). Based on these specifications, a sample size of 55 participants is required to detect a significant effect with low to medium effect sizes. To account for potential early drop-out and bad-quality EEG data, we increased the final sample size to 60 participants (10% estimated drop-out rate).

### Randomization and Blinding

We used block randomization with a total of 10 blocks, each containing *n* = 6 participants. The randomization sequence and study drugs were prepared by the pharmacy of the University Hospital Basel. All study personnel were blinded during data collection. We kept an unblinding list for safety purposes (in case of adverse events) that was only accessible to the study PI. We asked participants to guess which substance (placebo, haloperidol, L-DOPA, or “I cannot say”) they had received at the end of each session.

### EEG Recording and Preprocessing

The EEG recording was conducted with 64 electrodes according to the international 10–10 system, which were mounted on an elastic cap (BioSemi ActiveTwo system). The sampling rate was 1024 Hz, and electrode impedances were kept below 25 kΩ. Eye movements were recorded using four EOG channels attached horizontally at the right and left temple and vertically below and above the left eye. Ten minutes of eyes-closed resting-state data were recorded while participants were seated in a light-shielded, quiet room. To prevent drowsiness, participants were asked to open their eyes every two minutes for 10 s when prompted by an audio cue over headphones.

The EEG data were preprocessed offline using Brain Vision Analyzer (Version 2.2.2, Brain Products GmbH, Munich, Germany). We filtered the raw data with an IIR bandpass filter between 0.5 and 70 Hz, combined with a 50 Hz notch filter. Severe artifacts caused by movement or poor signal quality were removed upon visual inspection. EEG channels with poor quality across the entire recording were interpolated (max 10%), and eye movements were removed using extended infomax ICA (max 10%). The data were segmented into 2-second epochs, and epochs with remaining artifacts were removed using semi-automatic artifact rejection (maximal gradient: 50 µV/ms, Max-Min difference: 200 µV, low activity: 0.5 µV). We down-sampled the data to 256 Hz and re-referenced to the average reference. Only eyes-closed data were used for the microstate analysis.

### Microstates Analysis

We performed the microstate analysis using Matlab (R2021b), eeglab (v2022.0) and the MICROSTATELAB toolbox (version 1.0) (Nagabhushan Kalburgi et al. 2024). The preprocessed EEG data were filtered between 2 and 20 Hz. First, we identified individual microstate topographies at global field power peaks using modified k-means clustering (50 restarts). The number of clusters was set to k = 4–7. Next, we used the individual topographies belonging to the drug conditions to calculate mean microstate topographies for each condition. The condition means were then used to calculate grand mean topographies. The grand mean topographies were sorted and class-labeled according to template maps available in the toolbox (Zanesco et al. 2021). We used the grand mean topographies to sort the condition mean topographies. We compared the grand mean and condition mean topographies to other published studies, and found that 5 classes were corresponding most with previously reported mean maps (Tarailis et al. 2023). In the backfitting procedure, all individual topographies were assigned to the best-fitting condition mean template topographies. The following parameters were extracted and served as the primary outcome measures: the average contribution of each class to the entire recording duration (coverage), the average duration in milliseconds (duration), and the average number of occurrences per second (occurrence). In addition to the classical microstates parameters, we also examined transitions using Markov Models (see Supplementary Material for more details).

### Secondary Outcomes

Several secondary outcomes were assessed on each study day: (i) *Subjective effects of substance.* We assessed the subjective psychological, somatic, and motor effects of the substances at three different time points: before ingesting the first capsule (baseline), after ingesting the second capsule, and at the end of the visit. The questionnaire consisted of 21 items that were rated on a 1–4-point Likert-scale, a higher score indicating stronger subjective effects. Further secondary outcomes were assessed directly after the EEG recording: (ii) Using the *Community Assessment of Psychic Experiences (CAPE42)* (Mossaheb et al. 2012), we assessed (sub)psychotic experiences. The CAPE42 is a 42-item self-report questionnaire that yields scores for positive, negative, and depressive symptoms. (iii) Nonspecific effects of the substances on attention were assessed with the *d2-test* (Ross 2005*)*, a test of selective attention. (iv) The *New York Cognition Questionnaire (NYC-Q)* was used to assess the content (positive, negative, future, past, friends) and form (images, words, vague/specific) of an individual’s thoughts during the acquisition of the EEG (Gorgolewski et al. 2014).

### Statistical Methods

We used linear mixed models, implemented using the ‘lmerTest’ package in the R statistical environment (version 4.3.2) and RStudio (version 2023.12.0 + 369), to examine the effects of each drug condition on the EEG microstate parameters and secondary outcomes. We fitted a linear mixed model (estimated using REML) on each microstate parameter to predict each outcome measure (coverage, duration, occurrence) with class and condition. The model included Participant ID as random effect. We then performed ANOVAs (Satterthwaite’s method ) on the model to assess main and interaction effects. Significant interactions were followed by mixed linear models for each class separately followed by paired t-tests for pairwise comparisons between conditions. Reported effect sizes are Cohen’s d. We used a Pearson’s chi-square test to assess blinding. Statistical tests were carried out two-sided and the statistical level was set at α = 0.05. P-values for post-hoc tests were corrected for multiple comparisons. Only participants with at least one analyzed EEG dataset were included in the final analysis.

## Results

### Participants

**Fig. 1:**
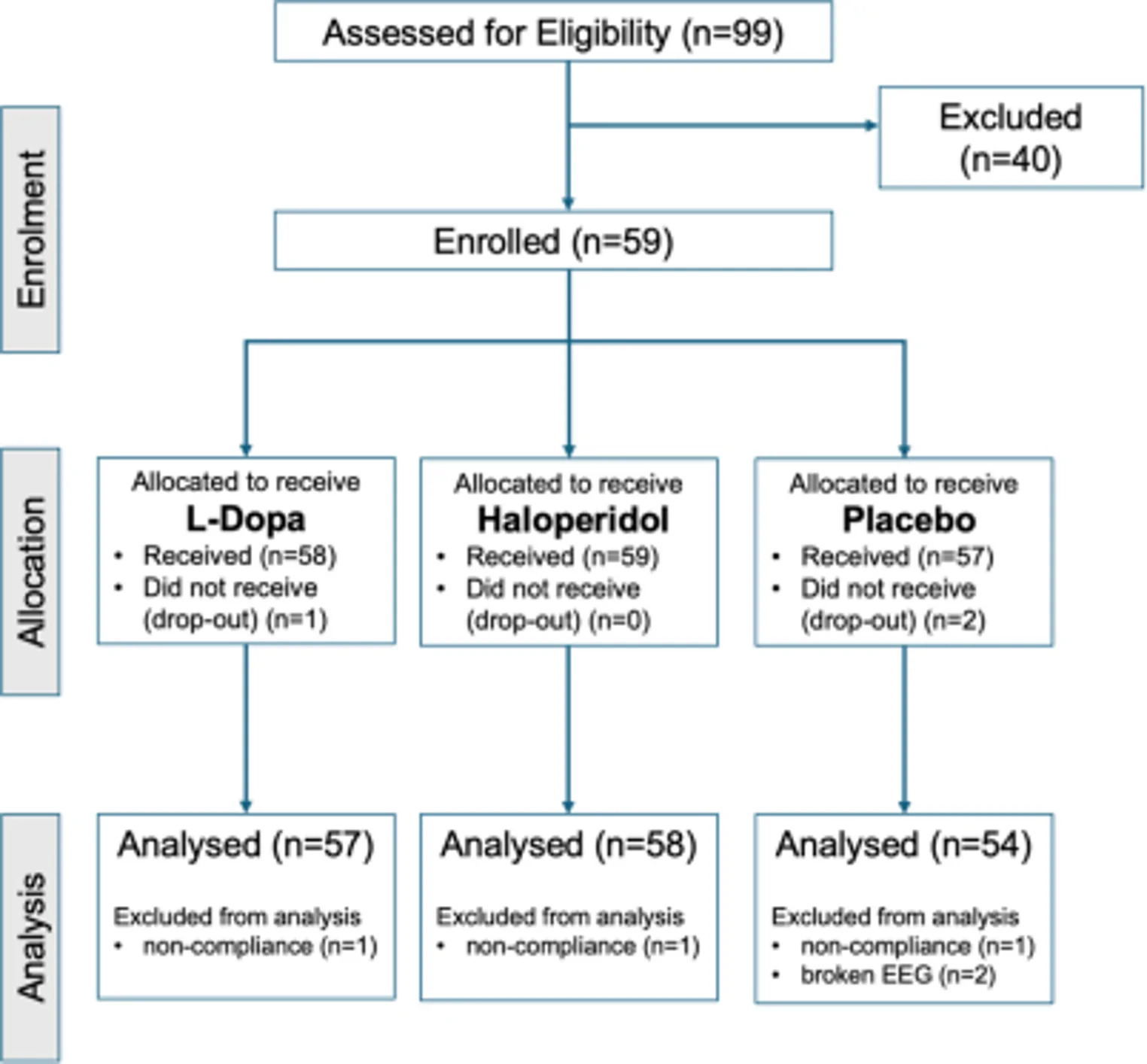
The CONSORT flowchart of participants recruited, enrolled, allocated and included in the analysis. We used a randomized, double-blind, cross-over, and placebo-controlled design

A total of 99 participants were assessed for eligibility. Of those, 59 were enrolled and randomized in the study. One participant dropped-out after the first visit and one person after the second visit. In addition, two EEG datasets were not recorded due to technical issues. One participant was excluded after data collection because of non-compliance with the study protocol. This resulted in a final sample of 58 participants, with a total of 169 EEG datasets analyzed, consisting of 58 haloperidol, 57 L-DOPA, and 54 placebo datasets (Fig. 1). The final sample consisted of 26 female and 32 male participants, with a mean age of 25.10 years (*SD* = 4.56, range = 18–39 years) (Table 2). Blinding to the substances was preserved as we found no significant relationship between the ingested and guessed substances (*X*^2^(2) = 3.30, *P* = 0.192).

**Table 2:** Participant demographics

Variable	*N* = 58
*Sex*,* n (%)*	
Female	26 (45%)
Male	32 (55%)
*Age*,* Mean (SD)*	25.1 (4.6)
*Handedness*,* n (%)*	
Left	7 (12%)
Right	51 (88%)
*School years*,* Mean (SD)*	12.13 (2.52)
Unknown	3
*Further education*,* n (%)*	
No degree	23 (40%)
Apprenticeship	5 (8.8%)
University of Applied Sciences	3 (5.3%)
University	26 (46%)
Unknown	1
*Civil status*,* n (%)*	
Single	43 (74%)
Relationship	12 (21%)
Married	3 (5.2%)
*Employment*, *n (%)*	36 (62%)
*Alcohol use*, *n (%)*	42 (72%)
*Nicotine use*, *n (%)*	7 (12%)
*Cannabis use*,*n (%)*	1 (1.7%)

### Microstates

**Fig. 2:**
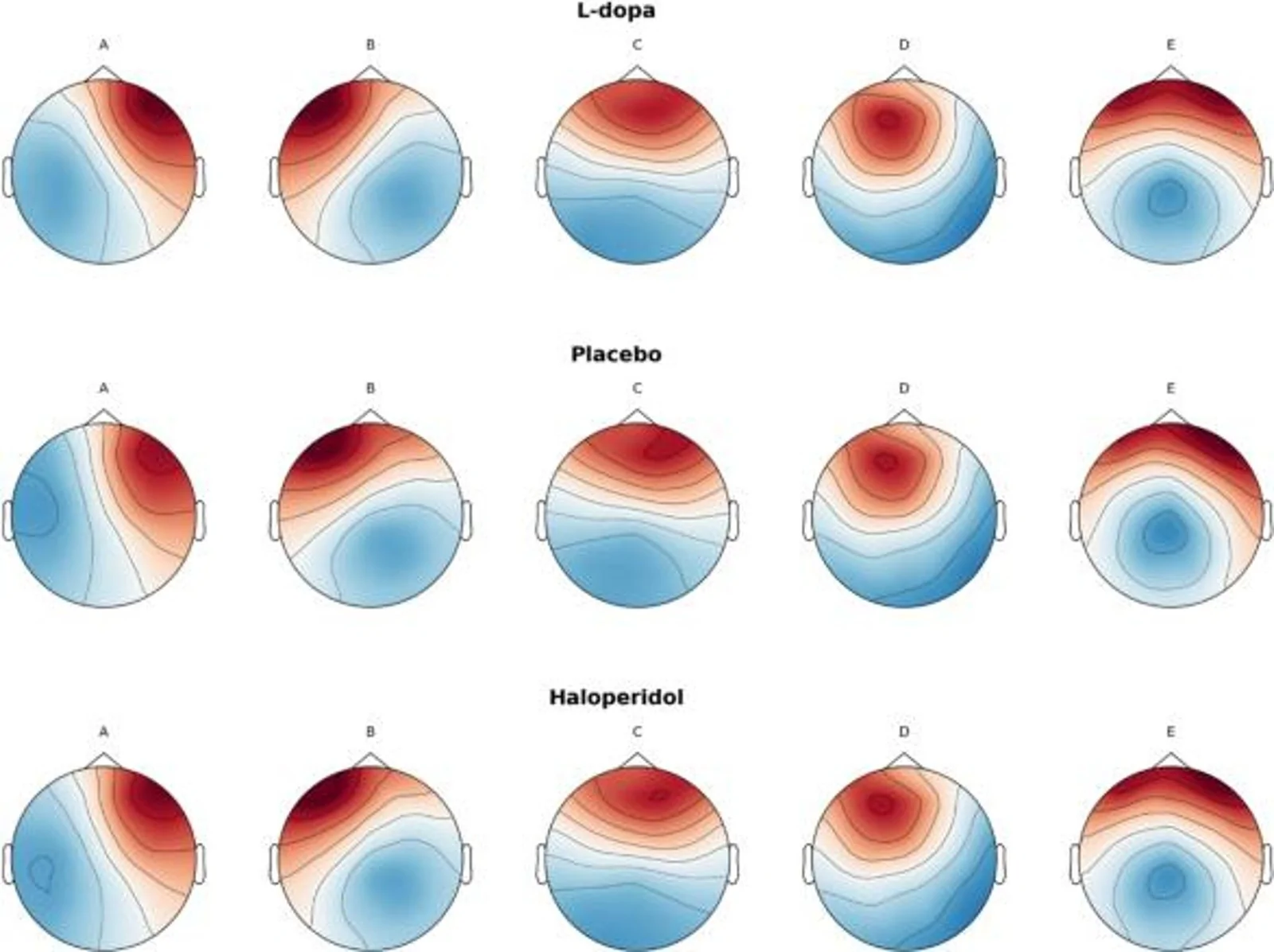
Canonical microstate topographies for each condition

Figure 2 displays the five microstate topographies for each condition. The explained variance of the group fitting was 69% on average in all conditions and did not significantly differ between conditions ($\:F\left(2,109.19\right)=0.09$, * p=.917*). In addition, the mean total time (that had a microstate class assigned) did not significantly differ between conditions ($\:F\left(2,110.89\right)=1.17$, * p=.316*) and averaged 381.7 s (*SD* = 69.1, range = 136–469 s).

**Fig. 3:**
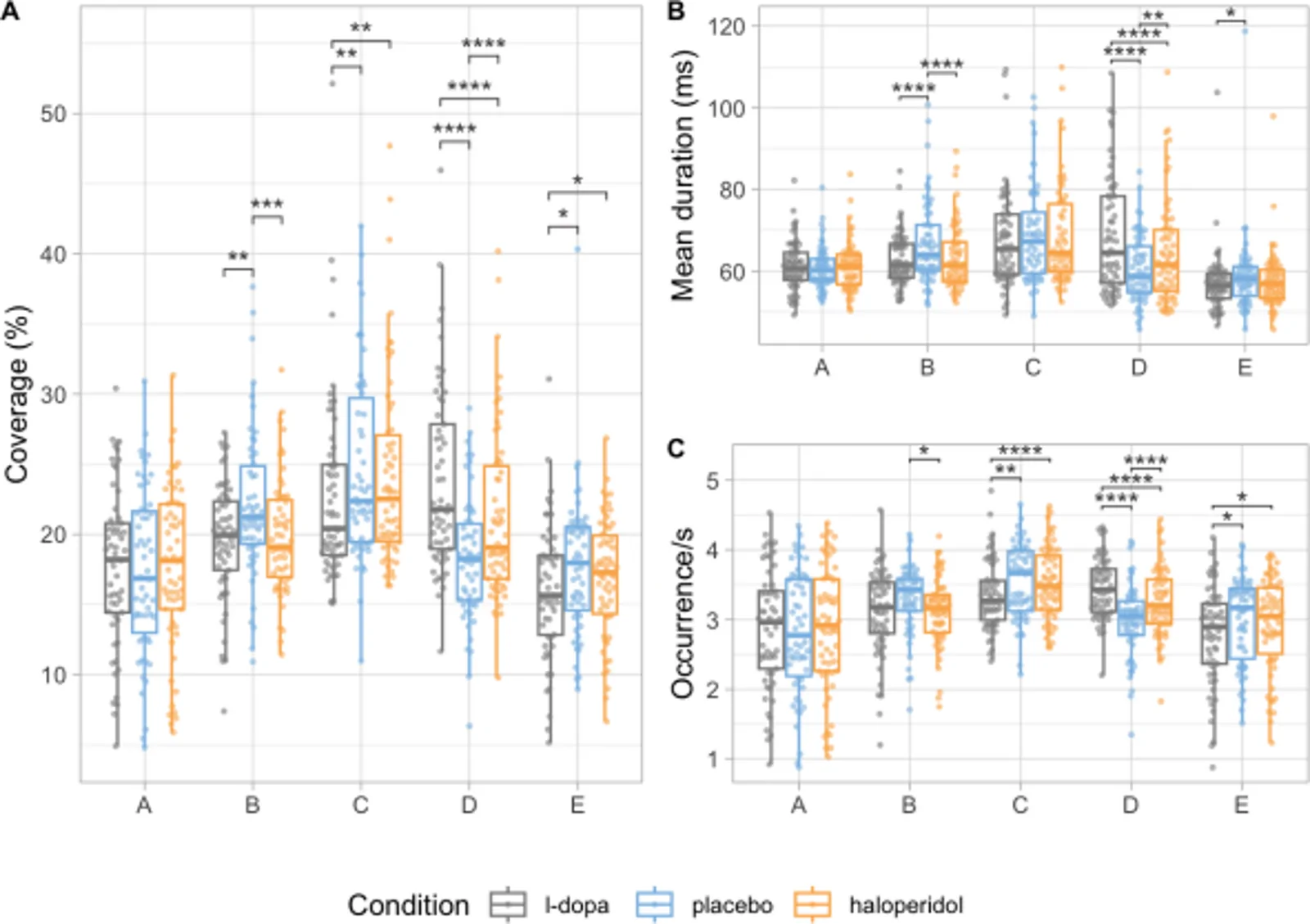
Microstates statistics. Plots show boxplots of each condition and microstate class show median and IQR, and individual datapoints of the temporal parameters: (**A**) coverage, (**B**) duration and (**C**) occurrence. P values are Holm corrected (15 comparisons per parameter) *p < 0.05, **p < 0.01, ****p < 0.0001

Significant condition-by-class interactions were observed for all parameters: coverage: $\:F\left(8,830\right)=4.95$, * p<.001*; duration: $\:F\left(8,772.91\right)=4.83$, * p<.001*; occurrence: $\:F\left(8,772.79\right)=3.82$, * p<.001*). Compared to placebo, microstate B was decreased in L-DOPA and haloperidol. Parameters of microstate C and E were reduced under L-DOPA compared to haloperidol and placebo. Both haloperidol and L-DOPA increased parameters of microstate D, compared to placebo. Figure 3 shows the violin plots for coverage, duration and occurrence. Table 3 displays all means and SDs for coverage, duration and occurrence, and the results of the post-hoc pairwise comparisons.

**Table 3:** Mean values and standard deviations for each condition and each microstate parameter. Statistical analysis results: overall repeated measures ANOVA, and W, P values and effect size Cohen’s d for each post-hoc pairwise comparison. Post-hoc *P* values are Holm corrected (15 comparisons per parameter)

		L-DOPA	Placebo	Haloperidol	ANOVA		L-DOPA vs. Placebo			Placebo vs. Haloperidol			L-DOPA vs. Haloperidol		
		*n* = 57	*n* = 54	*n* = 58	F	p	t	p	d	t	p	d	t	p	d
Coverage	MS A	17.7 (5.63)	17.3 (5.98)	17.7 (5.83)	0.714	0.492	–	–	–	–	–	–	–	–	–
	MS B	19.5 (4.19)	22.1 (5.38)	19.9 (4.14)	12.355	< 0.001	-3.597	**0.001**	-0.49	4.027	**0.001**	0.55	-0.864	0.391	-0.11
	MS C	22.9 (6.76)	24.6 (6.70)	24.3 (6.91)	5.746	0.004	-2.988	**0.009**	-0.41	0.49	0.626	0.07	-3.514	**0.003**	-0.47
	MS D	23.8 (6.63)	18.3 (4.54)	21.2 (6.13)	43.878	< 0.001	8.321	**< 0.001**	1.13	-4.539	**< 0.001**	-0.62	5.406	**< 0.001**	0.72
	MS E	16.0 (4.78)	17.8 (5.08)	17.0 (4.25)	5.625	0.005	-3.008	**0.012**	-0.41	1.096	0.278	0.15	-2.485	**0.032**	-0.33
Duration	MS A	61.2 (6.18)	60.7 (5.32)	61.3 (6.07)	0.188	0.829	–	–	–	–	–	–	–	–	–
	MS B	62.9 (6.47)	67.0 (10.4)	63.6 (8.08)	18.081	< 0.001	-4.673	**< 0.001**	-0.64	4.94	**< 0.001**	0.67	-1.254	0.215	-0.17
	MS C	67.8 (12.4)	69.1 (12.0)	68.7 (12.5)	1.897	0.155	–	–	–	–	–	–	–	–	–
	MS D	68.6 (14.3)	60.6 (8.29)	64.6 (12.9)	25.242	< 0.001	6.888	**< 0.001**	0.94	-3.086	**0.003**	-0.42	4.038	**< 0.001**	0.53
	MS E	57.2 (7.84)	58.9 (9.75)	57.7 (7.40)	3.363	0.038	-2.568	**0.039**	-0.35	1.415	0.326	0.19	-1.055	0.326	-0.14
Occurrence	MS A	2.87 (0.844)	2.82 (0.888)	2.87 (0.894)	1.020	0.364	–	–	–	–	–	–	–	–	–
	MS B	3.11 (0.627)	3.29 (0.496)	3.12 (0.500)	4.589	0.012	-2.181	0.067	-0.3	2.571	**0.039**	0.35	-0.269	0.789	-0.04
	MS C	3.35 (0.495)	3.53 (0.539)	3.52 (0.515)	10.200	< 0.001	-3.601	**0.001**	-0.49	0.117	0.907	0.02	-4.675	**< 0.001**	-0.62
	MS D	3.45 (0.429)	2.98 (0.535)	3.25 (0.494)	37.480	< 0.001	7.316	**< 0.001**	1.00	-4.706	**< 0.001**	-0.64	4.857	**< 0.001**	0.64
	MS E	2.78 (0.717)	2.99 (0.634)	2.93 (0.671)	5.718	0.004	-2.597	**0.024**	-0.35	0.372	0.712	0.05	-2.872	**0.017**	-0.38

### First-order Markovian Analysis

**Fig. 4:**
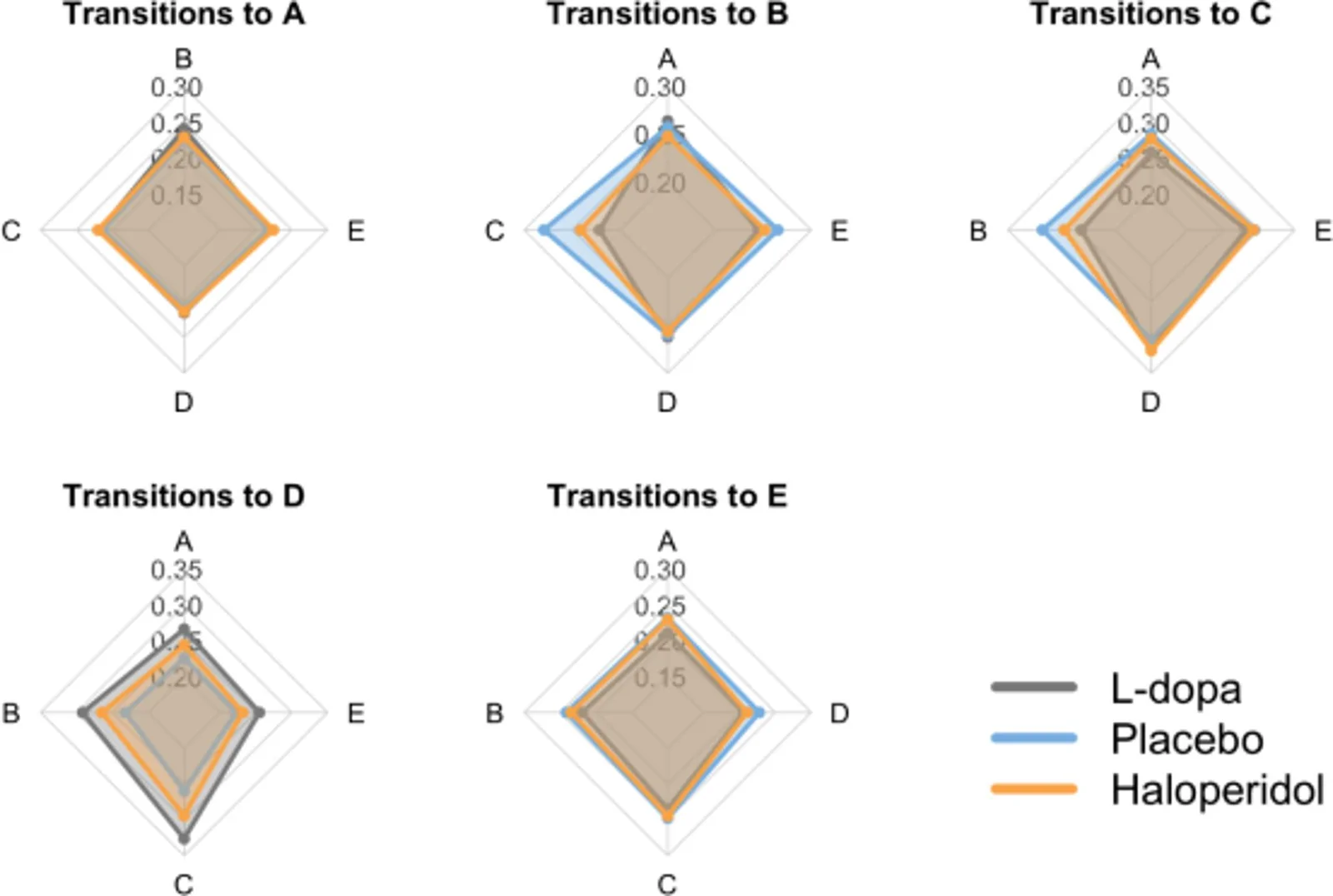
Radar chart showing the transitions to each microstate class from all microstates classes

In Supplementary Table 1 the matrices $\:S,\:P,\:{P}^{\left(0\right)},\:{X}^{2}$are presented for the statistically significant tests. Figure 4 shows the values of the probability of transition to each microstate class. Based on chi-square statistics with 16 degrees of freedom at * a=*0.05 significance level the matrix P is significantly different from $\:{P}^{\left(0\right)}$ when the values of $\:{X}^{2}$ is larger than 26.296.

### Secondary Outcomes

The questionnaire assessing subjective psychological, somatic, and motor effects was generally rated very low, indicating no strong subjective effects. The average score across time points and conditions was 23.2 (*SD* = 0.5, range = 21–35). We observed significant main effects of condition ($\:F\left(2,447.63\right)=4.52$, * p=.011*) and time point ($\:F\left(2,446.31\right)=11.84$, * p<.001*), with higher average scores on later timepoints and in the L-DOPA condition, but there was no significant interaction of condition*time point ($\:F\left(4,446.31\right)=0.68$, * p=.603*).

The CAPE-42 measures (sub)psychotic experiences on three subscales: positive, negative and depressive symptoms. Positive, negative, and depressive symptoms did not significantly differ between conditions (all *p* values > 0.05).

On the d2 test of attention, the average number of items crossed was 559.3 (*SD* = 70.4), and the average number of errors was 17.9 (*SD* = 16.5). Both scores did not differ between conditions (items: $\:F\left(2,106.79\right)=1.34$, * p=.266*; errors: $\:F\left(2,106.79\right)=1.34$, * p=.266*).

On the New-York Cognition Questionnaire, we found significant main effects of content ($\:F\left(4,772.95\right)=334.87$, * p<.001*) and form ($\:F\left(2,441.68\right)=93.48$, * p<.001*). There were no significant main effects of condition for either content ($\:F\left(2,777.91\right)=1.01$, * p=.363*) or form ($\:F\left(2,446.09\right)=0.32$, * p=.726*) and no significant interactions (content: $\:F\left(8,772.95\right)=0.32$, * p=.960*; form: $\:F\left(4,441.68\right)=0.68$, * p=.607*).

## Discussion

The aim of this study was to investigate the acute effects of dopaminergic drugs on resting-state EEG microstates in healthy participants. We used haloperidol, a dopamine D2 receptor antagonist and common antipsychotic, to simulate effects of antipsychotic treatment by reducing dopaminergic transmission. To also assess the effects of increased dopaminergic transmission, we used L-DOPA (a dopamine precursor). As expected, we observed that both dopaminergic drugs affect various resting-state EEG microstates classes and parameters, both in a linear and non-linear (U-shaped) way. However, the direction of effects was not always consistent with a clinical perspective. We did not find any statistically significant drug effects on the secondary outcome measures.

Duration, occurrence, and coverage of microstate class A were numerically increased in both drug conditions compared to placebo; however, this effect was not significant. Both drugs showed significantly increased transitions towards class A. Meta-analyses do not report any differences in microstate A comparing patients and controls (da Cruz et al. 2020; Rieger et al. 2016; Vass et al. 2025). However, one study found an increase of microstate A in a combined sample of first-episode psychosis and high-risk patients compared to healthy controls (de Bock et al. 2020), and a meta-analysis including patients with anxiety disorders also observed an increase of microstate A in patients (Chivu et al. 2024). The pattern of results indicates that alterations in microstate A might relate to general features of psychopathology, rather than specific psychotic symptoms or dopamine function.

Microstate class B showed decreased temporal parameters both in the L-DOPA and the haloperidol condition (compared to placebo), indicating an inverted-U shape, non-linear, relationship between dopamine activity and microstate B. As mentioned before, non-linear effects of dopamine have been associated with cognitive processes (Cools and D’Esposito 2011). This interpretation is further supported by findings linking microstate B to cognitive domains (Tarailis et al. 2023). Therefore, the observed drug effects on microstate B are more likely to reflect cognitive effects and less likely to be relevant in the pathophysiology and/or treatment of positive symptoms. Indeed, in patients with psychotic disorders, meta-analyses report no significant group differences between patients and healthy participants in microstate B (da Cruz et al. 2020; Rieger et al. 2016; Vass et al. 2025).

In microstate class C, D, and E, our results are not completely in line with what we expected from previous studies in patients with psychotic disorders. For microstate D, previous research and meta-analysis have reported reduced coverage and duration in patients with psychotic disorders (da Cruz et al. 2020; Rieger et al. 2016), which have been suggested to be driven by unmedicated patients (Vass et al. 2025). In addition, one study observed an increase in microstate D parameters in patients medicated with antipsychotics compared to healthy controls (Baradits et al. 2020). Taken together, these findings suggest an inverse linear relationship between dopamine activity and class D parameters. Instead, we observed a non-linear, U-shaped relationship with increased coverage, longer duration, and more occurrences of microstate D, and more transitions towards this microstate class, in both drug conditions compared to placebo. Moreover, in microstate class C, we observed reduced coverage and fewer occurrences in the L-DOPA condition, compared to placebo and haloperidol. In contrast, coverage and occurrence of microstate C are increased in patients with psychotic disorders (da Cruz et al. 2020; Rieger et al. 2016; Vass et al. 2025). In both cases, deviations may be because our single-dose paradigm was not able to reproduce all aspects of microstate changes associated with psychotic disorders. For example, a meta-analysis by Vass et al. (2025) demonstrated significant differences in microstate parameters C and D depending on medication status and illness progression stage. More fundamentally, although L-DOPA increases dopamine levels, it cannot reproduce the specific and complex pattern of dopaminergic dysregulation in psychotic disorders.

It should be pointed out that the optimal number of microstate classes remains a topic of debate and may influence comparability across studies. In this study, we chose a five-class model, based on an increasing number of studies now optimizing class number beyond the conventional four classes. Literature on microstates in schizophrenia still predominantly relies on a four-class model. However, recent studies have drawn attention to the ambiguity of microstate class C within this model, as its topography might rather correspond to microstate E (Tarailis et al. 2023). Still, the topography of class C shows high spatial correlation with microstate class E in five-class solutions. One should therefore be cautious interpreting and comparing the results of microstate C and E with other studies, even though both microstate classes showed a similar pattern of response to dopaminergic agents in our study.

The within-subject design of this study minimized interindividual variability and increased the sensitivity to detect dopaminergic effects. In addition, pharmacokinetic variability was controlled through standardized dietary instructions and fixed timing between drug intake and EEG recordings. However, as already noted, single-dose pharmacological challenges in healthy participants cannot fully capture the chronic dopaminergic and neuroplastic adaptations that develop during psychotic disorders and long-term antipsychotic treatment. In addition, in clinical practice, patients often receive different types of antipsychotics. Future research should include several different antipsychotics to study longitudinal effects in patients.

## Conclusion

In the present study, we used a randomized, double-blind, cross-over, placebo-controlled design to examine the effects of dopaminergic modulation on resting-state EEG microstates in healthy participants. We demonstrate that dopaminergic drugs alter microstate parameters in both linear and non-linear (U-shaped) ways, indicating that dopamine contributes to large-scale network dynamics. However, extrapolations from healthy volunteers to (medicated) patients with psychotic disorders are limited by different factors such as illness progression, long-term medication, and complex patterns of dopaminergic dysregulation. For this reason, drug-challenge studies cannot reproduce patient findings directly but instead offer a controlled framework for isolating neurotransmitter-specific effects. Taken together, our findings highlight the importance of further drug-challenge research in healthy participants, as well as research into the effects of different dopaminergic and antipsychotic medications in clinical populations.

## Supplementary Information

Below is the link to the electronic supplementary material.

## Data Availability

Data supporting the findings of this study are available from the corresponding author upon reasonable request.

## Declarations

### AI Statement

Generative AI (ChatGPT-5, OpenAI) was used to assist with language editing, including improving clarity, grammar, and overall readability of the English text. The authors reviewed and approved all generated content and take full responsibility for the final version of the manuscript.
