# Supplementary material for: Dopaminergic Drugs Modulate Resting-State EEG Microstates in Healthy Participants
Source: Brain Topogr. 2026 Jul 15;39(5):79. doi: 10.1007/s10548-026-01232-4 (PMC13373011; doi:10.1007/s10548-026-01232-4)
Supplement: Supplementary file 1 — Supplementary Material 1 [file 10548_2026_1232_MOESM1_ESM.docx]

Dopaminergic drugs modulate resting-state EEG microstates in healthy participants

Renate de Bock^1*^ , https://orcid.org/0000-0003-3774-2086

Amatya J. Mackintosh^2^, https://orcid.org/0000-0002-4535-8829

Alexandra Korda^3,4^, https://orcid.org/0000-0001-8843-4951

Alina Preuss^3,4^, https://orcid.org/0009-0004-4254-7247

Daniel J. Hauke^5^, https://orcid.org/0000-0003-1772-9239

Andreea O. Diaconescu^6,7^, https://orcid.org/0000-0002-3633-9757

Philipp Sterzer^1^, https://orcid.org/0000-0003-4687-2317

Stefan Borgwardt^3,4^, https://orcid.org/0000-0002-5792-3987

Christina Andreou^3,4^, https://orcid.org/0000-0002-6656-9043

^1^ Department of Psychiatry (UPK), University of Basel, Wilhelm Klein-Strasse 27, 4002, Basel, Switzerland

^2^ Faculty of Psychology, Department Clinical Psychology and Epidemiology, Missionsstrasse 62a, 4055 Basel, Switzerland

^3^ Translational Psychiatry, Department of Psychiatry and Psychotherapy, University of Lübeck, Ratzeburger Allee 160, 23562, Lübeck, Germany

^4^ Center of Brain, Behavior, and Metabolism (CBBM), University of Lübeck, Ratzeburger Allee 160, 23562, Lübeck, Germany

^5^ Hawkes Institute, Department of Computer Science, University College London, 90 High Holborn, London WC1V 6LJ, United Kingdom

^6^ Krembil Centre for Neuroinformatics, Centre for Addiction and Mental Health, 250 College St, Toronto, ON M5T 1R8

^7^ Department of Psychiatry, University of Toronto, Toronto, Canada

*Correspondence concerning this article should be addressed to Renate de Bock, [renate.debock@unibas.ch](mailto:renate.debock@unibas.ch)

# Methods

In addition to the classical microstates parameters, we also examined transitions using Markov Models. The state transition matrices (STMs) 𝑆 tabulate the cases of transitions that occurred between states (microstates in our case). *S_ij_* is calculated as

𝑆_𝑖𝑗_=𝑛_𝑖𝑗_

where the number of transitions from the microstate class 𝑖 to microstate class 𝑗 is referred as 𝑛_𝑖𝑗_.

Quantifying incidence dynamics in microstates successions requires the calculation of state transition probability matrices (STPMs). The probability of transition of microstate class $i$ to microstate class $j , S_{ij}$,is calculated as

$$P_{ij}=\frac{n_{ij}}{n_{i}}$$

where the number of transitions from the microstate class $i$ to microstate class $j$ is referred as $n_{ij}$, and the number of transitions from microstate class $i$ to any state as $n_{i}$.

These state transitions probabilities are compared with the probability of being at microstate class $i$, $P_{ij}^{(0)}$, independently of the occurrence of the microstate class j.

$$P_{ij}^{(0)}=\frac{n_{i}}{n}$$

where $n$ is the number of the transitions occurred.

Comparison of transition probability matrices was implemented via hypothesis tests, based on $X^{2}$statistics, described by (Anderson & Goodman, 1957)

# Results

| **i,j state index** | $S_{ij}$ | $P_{ij}$ | $P_{ij}^{(0)}$ | $X^{2}$ | **i,j state index** | $S_{ij}$ | $P_{ij}$ | $P_{ij}^{(0)}$ | $X^{2}$ | **i,j state index** | $S_{ij}$ | $P_{ij}$ | $P_{ij}^{(0)}$ | $X^{2}$ |
| --- | --- | --- | --- | --- | --- | --- | --- | --- | --- | --- | --- | --- | --- | --- |
| **L-dopa** | | | | 8.81×10^8 | **Placebo** | | | | 8.18×10^8 | **Haloperidol** | | | | 8.60×10^8 |
| A 🡪 B | 16284 | 0.26 | 0.20 |  | A 🡪 B | 14722 | 0.26 | 0.21 |  | A 🡪 B | 15094 | 0.25 | 0.20 |  |
| A 🡪 C | 15910 | 0.26 | 0.22 |  | A 🡪 C | 16228 | 0.28 | 0.23 |  | A 🡪 C | 16886 | 0.28 | 0.23 |  |
| A 🡪 D | 16420 | 0.27 | 0.22 |  | A 🡪 D | 12891 | 0.23 | 0.19 |  | A 🡪 D | 14852 | 0.24 | 0.21 |  |
| A 🡪 E | 12995 | 0.21 | 0.18 |  | A 🡪 E | 13266 | 0.23 | 0.19 |  | A 🡪 E | 13988 | 0.23 | 0.19 |  |
|  |  |  |  |  |  |  |  |  |  |  |  |  |  |  |
| B 🡪 A | 16201 | 0.24 | 0.18 |  | B 🡪 A | 15190 | 0.23 | 0.18 |  | B 🡪 A | 15339 | 0.23 | 0.18 |  |
| B 🡪 C | 16622 | 0.25 | 0.22 |  | B 🡪 C | 20363 | 0.30 | 0.23 |  | B 🡪 C | 18135 | 0.27 | 0.23 |  |
| B 🡪 D | 19555 | 0.29 | 0.22 |  | B 🡪 D | 15659 | 0.23 | 0.19 |  | B 🡪 D | 17675 | 0.26 | 0.21 |  |
| B 🡪 E | 14732 | 0.22 | 0.18 |  | B 🡪 E | 16287 | 0.24 | 0.19 |  | B 🡪 E | 15658 | 0.23 | 0.19 |  |
|  |  |  |  |  |  |  |  |  |  |  |  |  |  |  |
| C 🡪 A | 15948 | 0.22 | 0.18 |  | C 🡪 A | 15633 | 0.21 | 0.18 |  | C 🡪 A | 16587 | 0.22 | 0.18 |  |
| C 🡪 B | 16259 | 0.22 | 0.20 |  | C 🡪 B | 20485 | 0.28 | 0.21 |  | C 🡪 B | 18179 | 0.24 | 0.20 |  |
| C 🡪 D | 23954 | 0.33 | 0.22 |  | C 🡪 D | 19046 | 0.26 | 0.19 |  | C 🡪 D | 22185 | 0.29 | 0.21 |  |
| C 🡪 E | 17224 | 0.23 | 0.18 |  | C 🡪 E | 18165 | 0.25 | 0.19 |  | C 🡪 E | 18458 | 0.24 | 0.19 |  |
|  |  |  |  |  |  |  |  |  |  |  |  |  |  |  |
| D 🡪 A | 16278 | 0.22 | 0.18 |  | D 🡪 A | 12836 | 0.21 | 0.18 |  | D 🡪 A | 14825 | 0.21 | 0.18 |  |
| D 🡪 B | 19761 | 0.26 | 0.20 |  | D 🡪 B | 15943 | 0.26 | 0.21 |  | D 🡪 B | 17766 | 0.26 | 0.20 |  |
| D 🡪 C | 23772 | 0.32 | 0.22 |  | D 🡪 C | 18609 | 0.30 | 0.23 |  | D 🡪 C | 22061 | 0.32 | 0.23 |  |
| D 🡪 E | 15537 | 0.21 | 0.18 |  | D 🡪 E | 13960 | 0.23 | 0.19 |  | D 🡪 E | 14576 | 0.21 | 0.19 |  |
|  |  |  |  |  |  |  |  |  |  |  |  |  |  |  |
| E 🡪 A | 13182 | 0.22 | 0.18 |  | E 🡪 A | 13448 | 0.22 | 0.18 |  | E 🡪 A | 14068 | 0.22 | 0.18 |  |
| E 🡪 B | 14807 | 0.24 | 0.20 |  | E 🡪 B | 16350 | 0.27 | 0.21 |  | E 🡪 B | 15768 | 0.25 | 0.20 |  |
| E 🡪 C | 17080 | 0.28 | 0.22 |  | E 🡪 C | 18129 | 0.29 | 0.23 |  | E 🡪 C | 18327 | 0.29 | 0.23 |  |
| E 🡪 D | 15419 | 0.25 | 0.22 |  | E 🡪 D | 13752 | 0.22 | 0.19 |  | E 🡪 D | 14516 | 0.23 | 0.21 |  |

**Supplemental Table 1.** The table shows that the values of $P_{ij}$are significantly higher than $P_{ij}^{(0)}$ in all conditions, according to $X^{2}$ statistic. These results indicate that the transitions between the 5 microstate classes are deterministic. The generation of the microstates in resting-state EEGs is not random process but it appears to have “memory”. When comparing the $P_{ij}$ between the groups, there are transitions that can be characterised as equilibrium points in the transient space, independent of the microstate that the attractor starts will result to the same equilibrium point (e.g., microstate A). These attractors are stronger in some conditions compared to the others
